# A newly identified secreted larval antigen elicits basophil-dependent protective immunity against *N. brasiliensis* infection

**DOI:** 10.3389/fimmu.2022.979491

**Published:** 2022-08-25

**Authors:** Natalie Thuma, Daniela Döhler, Dirk Mielenz, Heinrich Sticht, Daniel Radtke, Lena Reimann, Bettina Warscheid, David Voehringer

**Affiliations:** ^1^ Department of Infection Biology, University Hospital Erlangen, Friedrich-Alexander Universität Erlangen-Nürnberg (FAU), Erlangen, Germany; ^2^ Division of Molecular Immunology, Department of Internal Medicine 3, University Hospital Erlangen, Friedrich-Alexander Universität Erlangen-Nürnberg (FAU), Erlangen, Germany; ^3^ Institute of Biochemistry, Friedrich-Alexander Universität Erlangen-Nürnberg (FAU), Erlangen, Germany; ^4^ Institute of Biology II, Biochemistry and Functional Proteomics, Faculty of Biology, University of Freiburg, Freiburg, Germany; ^5^ Department of Biochemistry, Theodor Boveri-Institute, University of Würzburg, Würzburg, Germany

**Keywords:** hookworms, basophils, SCP/TAPS protein, CAP domain, immunization

## Abstract

Hookworms infect more that 400 million people and cause significant socio-economic burden on endemic countries. The lack of efficient vaccines and the emergence of anthelminthic drug resistance are of major concern. Free-living hookworm larvae infect their hosts *via* the skin and live as adult worms in the small intestine where they feed on host tissue and blood. Excretory/secretory (E/S) products, released by helminths as they migrate through their host, are thought to play a key role in facilitating infection and successful establishment of parasitism. However, E/S products can also elicit protective immune responses that might be harnessed for vaccine development. By performing Western blots with serum of *Nippostrongylus brasiliensis* (Nb) infected mice as a model for human hookworm infection, we identified a largely overlapping set of IgG1- and IgE-reactive antigens in E/S from infective L3 stage larvae. Mass spectrometry analysis led to the identification of a new protein family with 6 paralogues in the Nb genome which we termed Nb-LSA1 for “*Nippostrongylus brasiliensis* larval secreted protein 1”. The recombinantly expressed 17 kDa family member Nb-LSA1a was recognized by antibodies in the serum of Nb immune mice. Immunization of mice with Nb-LSA1a in alum elicited a strong IgG1 response but no detectable antigen-specific IgE. Most importantly, immunized mice were largely protected against a challenge Nb infection. This effect was dependent on the presence of basophils and occurred before the parasites reached the intestine. Therefore, basophils appear to play a critical role for rapid control of infection with L3 stage larvae in mice immunized with a single secreted larval protein. A better understanding of basophil-mediated protective immunity and identification of potent larval antigens of human hookworms could help to develop promising vaccination strategies.

## Introduction

About a quarter of the human world population is infected with helminths, especially in low economic countries with poor sanitary conditions. Hookworms alone account for more than 400 million infections and cause major socioeconomic problems in endemic countries (1). Hookworm infections can result in anemia, malnutrition and intellectual disability of children. Anthelmintic drugs such as mebendazole or pyrantel can be used to efficiently reduce the worm burden but reinfections rapidly occur after deworming. In addition, there is evidence for increasing drug resistance to some anthelmintics (2). Despite major research efforts, there are no vaccines available yet for any human helminth infection (3).

The major human hookworm species are *Necator americanus* and *Ancylostoma duodenale* which live as adult worms for up to 10 years in the lumen of the small intestine and feed on blood and host tissue. Hookworms infect their hosts as free-living L3 larval stage by penetration of the skin. Next, they reach the lung *via* the bloodstream, enter the alveolar space, get coughed up and swallowed, to finally reach the lumen of the small intestine. Here, they mature to adult worms, feed on host tissue and blood, and females produce eggs that are excreted to the environment where the L1-L3 larval stages develop. Hookworms secrete a huge variety of different proteins which are poorly characterized but likely facilitate entry and persistence in their hosts. Such secretomes contain mainly three categories of proteins: proteases and protease inhibitors, sperm-coating proteins/Tpx-1/Ag5/PR-1/Sc7 (SCP/TAPS) including Venom Allergen-Like (VAL) or Activation-associated Secreted Proteins (ASPs) (Pfam acc. no. PF00188), and proteins with domains of unknown function (4). SCP/TAPS proteins are often immunogenic and therefore include candidates for vaccine development (5).

Although vaccines are unlikely to eradicate hookworm infections, it has been calculated that in combination with anthelmintics they would reduce the disability adjusted life years (DALYs) about 6-fold in a 10 years time frame as compared to administration of anthelmintics alone (6). The development of an efficient hookworm vaccine remains a major challenge and requires detailed understanding of molecular and cellular events required for an efficient and protective immune response. Ideally, protective immunity should be achieved in the skin to prevent larval migration to the lung and intestine.

Infection of mice with *Nippostrongylus brasiliensis (Nb)* is widely used to investigate potential mechanisms of protective immunity against hookworms. *Nb* is genetically related to *N. americanus* and has a similar life cycle. Although more prevalent in rats, *Nb* has also been isolated from wild mice (*Mus musculus*) (7). *Nb* elicits a strong type 2 immune response during primary infection and promotes worm expulsion from the intestine within 10 days by a “weep-and-sweep” mechanism that requires IL-13-elicited activation of goblet cells and smooth muscle cells. During a secondary infection, most L3 larvae are trapped in the skin or lung and only few parasites reach the intestine (8). We and others could show that protective immunity against secondary infection is provided by antibodies, basophils and alternatively activated macrophages (9–14).

In this study we performed Western blots with immune serum of *Nb*-infected mice followed by mass spectrometry of secreted proteins from infective *Nb* L3 stage larvae to identify new antigens with the potential to elicit protective immunity against *Nb* infection. We identified a 17 kDa protein which belongs to a new subfamily of SCP/TAPS proteins. This Nb-LSA1a protein elicited a strong IgG1 response but no detectable IgE upon immunization of mice. Importantly, Nb-LSA1a immunized mice showed a strong reduction in adult worm and egg counts. This protective effect occurred before larval stages reached the lung and was not observed in basophil-deficient Mcpt8Cre mice. These findings indicate that antibodies against Nb-LSA1a and perhaps other cross-reactive antigens activate basophils and prevent larval transit from skin to the lung.

## Materials and methods

### Mice

Mcpt8Cre mice on C57BL/6 background were bred and maintained in the Franz-Penzoldt Center in Erlangen and kept under specific pathogen free conditions. In Mcpt8Cre mice basophils are specifically and constitutively deleted as a result of Cre toxicity (15). C57BL/6 mice were obtained from Charles River Laboratories.

### Ethics statement

Animal experiments were approved by the Local Government of Lower Franconia and performed in accordance with German animal protection law and European Union guidelines.

### Parasite infection and enumeration of eggs and worms

For *N. brasiliensis* (*Nb*) infection mice were subcutaneously (s.c.) injected with 500 L3 stage larvae as previously described (15). To assess parasite fecundity, fecal egg counts were determined on day 7 post infection (p.i.) using a modified MacMaster counting chamber. Worm burden in the lung was analyzed on day 2 p.i. by enumeration of larvae that migrated out of the harvested lung tissue.

### Preparation of *N. brasiliensis* antigens

For preparation of *Nb* excretory/secretory proteins (NES) from L3 stage larvae or adult worms, the larvae were collected from the culture plates (L3) or intestine of infected mice (adults) and washed extensively (PBS/PenStrep). For collection of NES-containing supernatants, 10,000 larvae/mL for L3 or 100 worms/mL for adults were cultured in 1% glucose in PBS for 48-72h in 24-well plates at 37°C and 5% CO_2_. NES was passed through a 0.2 µm filter and stored at -80°C until used. *N. brasiliensis* somatic extract (NEX) was prepared by homogenization of L3 larvae on ice with stainless steel beads in PBS (TissueLyser, Qiagen, Hilden, Germany) followed by centrifugation and recovery of supernatant.

### His-tagged Nb-LSA1a protein expression and purification

For expression of His-tagged Nb-LSA1a protein, fullength cDNA was cloned in pcDNA3.1 (+) C-HA vector (**Supplementary Figure S1**). Transient transfection of HEK293T cells was performed at a cell confluency of 70-90% using 20 µg plasmid and standard calcium phosphate transfection technique (250 mM CaCl_2_ and HEPES-buffered saline). Supernatant containing His-tagged Nb-LSA1a protein was stored at -20°C until Ni-NTA purification (HisPur™ Ni-NTA Spin Columns, Thermo Fisher Scientific, Waltham, MA). For immunization experiments purified Nb-LSA1a protein or collected supernatant was used as indicated.

### Mouse immunization

Female C57BL/6 or Mcpt8Cre mice were immunized with Nb-LSA1a (purified or supernatant), NES or control (buffer used for Ni-NTA purification of protein or supernatant from empty vector transfected HEK293T cells) by intraperitoneal injection (i.p.) with 200 µL Imject Alum (Thermo Fisher Scientific). Nb-LSA1a or NES protein was used at 5-10 µg/mouse for prime and 1 µg/mouse for boost immunizations. Immunizations were performed on day 0, then boosted on day 7, before infection with *Nb* on day 14.

### Sample processing for LC-MS/MS

NES samples (1-10 µg) were prepared in 5x Laemmli buffer without β-mercaptoethanol (non-reducing condition), heated (95°C, 5 min) and analyzed by SDS-PAGE. Following visualization of proteins using colloidal Coomassie Brilliant Blue, gel lanes were cut into 6 slices covering approx. the mass range between 10 to 95 kDa. Slices were washed and destained by alternatingly incubating them with 10 mM NH_4_HCO_3_ and 50% (v/v) acetonitrile (ACN)/10 mM NH_4_HCO_3_ (10 min at room temperature (RT) each). Cysteine residues were reduced (5 mM TCEP/10 mM NH_4_HCO_3_, 30 min at RT) and alkylated (50 mM 2-chloroacetamid/10 mM NH_4_HCO_3_; 30 min at RT) followed by proteolytic digestion of proteins using trypsin (60 ng per slice; overnight at 37°C). Peptides were eluted with 0.5% (v/v) trifluoroacetic acid (TFA)/50% (v/v) ACN, dried *in vacuo*, resuspended in 30 μl 0.1% TFA and desalted with in-house prepared STAGE tips prior to LC-MS analysis.

### LC-MS/MS analysis

Reversed-phase liquid chromatography-mass spectrometry was performed using the UltiMateTM 3000 RSLCnano system (Dionex LC Packings/Thermo Fisher Scientific, Dreieich, Germany) coupled online to a Q Exactive Plus (Thermo Fisher Scientific, Bremen, Germany) instrument. The UHPLC system was equipped with two C18 μ-precolumns (Ø 0.3 mm × 5 mm; PepMap, Thermo Fisher Scientific) and an Acclaim PepMap™ analytical column (ID: 75 μm x 500 mm, 2 μm, 100 Å, Dionex LC Packings/Thermo Fisher Scientific). Peptides eluting from the LC column were transferred to a fused silica emitter for electrospray ionization using a Nanospray Flex ion source with DirectJunctionTM adaptor (Thermo Fisher Scientific) and applying a spray voltage of 1.5 kV and a capillary temperature of 200°C. The MS instrument was externally calibrated using standard compounds and equipped with a nanoelectrospray ion source and a stainless steel emitter (Thermo Fischer Scientific). MS parameters were as follows: MS scan range, *m/z* 375–1,700; resolution, 70,000 (at *m/z* 200); target value, 3 x 10^6^ ions; max injection time, 60 ms; TOP12-higher-energy collisional dissociation of multiply charged peptides; NCE of 28%; target value of 1 x 10^5^, maximum injection time of 120 ms; dynamic exclusion time of 45 s.

### Bioinformatics

For this study, the MaxQuant 1.6.10.43 was used with the UniProt database for *Nippostrongylus brasiliensis*, Taxonomy ID 27835, (release 2020_05; 22636 protein entries). The precursor mass tolerance was set to 20 ppm for the first search and to 4.5 ppm for the main search. Trypsin was set as proteolytic enzyme (≤2 missed cleavages). Oxidation of methionine and acetylation of the protein N-terminus was allowed as variable modifications and cysteine carbamidomethylation as fixed modification. A false discovery rate (FDR) of 1% was applied on both peptide (on modified peptides separately) and protein lists. The mass spectrometry proteomics data have been deposited to the ProteomeXchange Consortium *via* the PRIDE (16) partner repository with the dataset identifier PXD035568.

### AlphaFold

The three-dimensonal structure of Nb-LSA1a was predicted using AlphaFold v2.0 (without homologous structure templates and using a selected portion of the BFD database) (17, 18). The prediction is colored by model confidence band and the accuracy of the AlphaFold model was scored as highly accurate with a predicted local distance difference (pLDDT>90) on a scale from 0 to 100.

### 1D and 2D gel electrophoresis and Western blot

NES and NEX samples (1-10 µg), purified Nb-LSA1a protein or supernatant of transfected cells was subjected to reducing and non-reducing SDS-PAGE using precast gels (10-12% Mini-PROTEAN TGX, Biorad, Hercules, CA) and blotted onto a PVDF membrane according to manufacturer´s instructions (Trans-Blot Turbo System, Biorad). Therefore, samples were prepared in Laemmli buffer containing either 5% (reducing) or no β-mercaptoethanol (non-reducing). Membranes were blocked in 5% milk powder in Tris-buffered saline (TBS) with 0.1% Tween-20 (TBST) overnight at 4°C, before being incubated with indicated mouse serum samples (1:10 dilution in 3% bovine serum albumin (BSA)/PBS) overnight at 4°C. After extensive washing in TBST, bound immunoglobulin was detected by incubation with HRP-conjugated anti-mouse IgG (Fcγ fragment specific, Jackson ImmunoResearch, Ely, UK), 1:5000 diluted in 5% milk powder/TBST for 1 h at RT. Alternatively, blots were incubated with rat anti-mouse IgE or rat anti-mouse IgG1 (SouthernBiotech, Birmingham, AL), 1:200 in 5% milk powder/TBST) for 2 h at RT, followed by HRP-conjugated goat anti-rat (Jackson ImmunoResearch), 1:5000 in 5% milk powder/TBST for 1 h at RT. For detection of the His-tag, the blot was incubated with polyclonal rabbit anti-His antibody (Cell Signaling, Danvers, MA), 1:1000 3% BSA/PBS for 2 h at RT. Detection followed by HRP-conjugated donkey anti-rabbit (Jackson ImmunoResearch, 1:5000 in 5% milk powder/TBST) and membrane was developed as above. For 2D SDS-PAGE, proteins are separated by isoelectric focusing (IEF) using precast gels (SERVAGel) prior to standard separation by size (SDS-PAGE). In contrast to standard SDS-PAGE, the used NES samples were desalted (Zeba spin columns, Thermo Fisher Scientific) and directly eluted in IEF sample buffer and loaded onto the gel. Subsequent western blotting was carried out as described above.

### Elisa

Detection of IgE and IgG1 levels in the serum of naïve and infected mice was determined as follows: Purified mouse anti-IgE (clone R35–72, BD Biosciences, Franklin Lakes, NJ) or a commercial IgG1 ELISA kit (SouthernBiotech) was used for coating. As secondary reagents anti-mouse IgE-AP or IgG1-AP (SouthernBiotech), followed by development with pNPP substrate (SouthernBiotech) was applied. For detection of parasite-specific IgE or IgG1, a 10-20 μg/mL NES protein suspension (**Supplementary Figure S2**) was coated on 96-well polystyrene plates overnight (4°C), blocked with 3% BSA/PBS for 2 h and then incubated for 2 h with serum dilutions. Parasite-specific antibodies were determined using the secondary reagents described above. For Nb-LSA1a-specific ELISA, 96-well polystyrene plates were coated with a 10-20 µg/mL Nb-LSA1a suspension. Absorption was measured at 405 nm on a Multiskan FC photometer (Thermo Fisher) and blank wells were used for background subtraction.

### Statistical analysis

Statistical analysis was performed with Sigmaplot (Version 12.3, Systat Software) using Mann-Whitney U-test. Data is always indicated as mean + standard error (SEM). Levels of significance: *p < 0.05, **p < 0.01. n.s. = not significant.

## Results

### Immune serum from secondary Nb-infected mice stains a discrete set of parasite-secreted antigens

Infective L3 stage larvae of *Nb* secrete a large variety of proteins and other molecules (collectively termed *Nippostrongylus brasiliensis* excretory/secretory products, NES) some of which may play a critical role for entry of L3 larvae into the host organism *via* the skin barrier and for successful establishment of parasitism within their hosts. We therefore reasoned that identification of immunogenic proteins in NES could help to develop a vaccination strategy and dissect the mechanisms of protective immunity against the early stage of infection in the skin.

**Figure 1:**
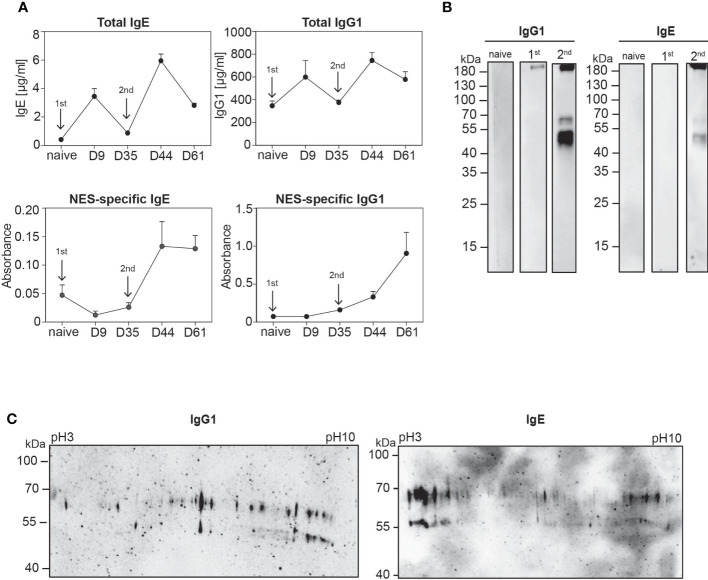
Polyclonal IgE and IgG1 antibodies from secondary *Nb-*infected mice recognise a discrete set of *Nb* antigens. **(A)** Graphs display kinetic of total (upper row) and NES-specific (lower row) serum IgE and IgG1 antibodies during the course of *Nb* infection, collected at indicated time points after primary (1^st^) and secondary (2^nd^) *Nb* infection (arrows) of wild-type mice or from naïve mice. Mean+SEM with 2-6 mice per group. **(B)** NES from L3 stage larvae were separated by standard SDS-PAGE under non-reducing conditions. Western blots were performed with serum from naïve, 1^st^ or 2^nd^ *Nb-*infected mice, following detection with either anti-mouse IgE or anti-mouse IgG1. **(C)** Representative 2D Western blots of NES hybridized with serum from mice after 2^nd^ *Nb* infection, followed by detection with either anti-mouse IgE or anti-mouse IgG1.

As a first step we determined total and NES-specific IgE and IgG1 levels in the serum after primary and secondary *Nb* infection of mice on C57BL/6 background. While total IgE and IgG1 levels increased after primary infection, we could not detect NES-specific IgE or IgG1 in the serum by ELISA (**Figure 1A**). This could be due to bystander activation of unspecific B cells or production of low-affinity antibodies. However, after secondary infection NES-specific IgE and IgG1 antibodies were clearly detectable by ELISA (**Figure 1A**). Next, we performed Western blot analysis. There was no antigen-specific IgG1 or IgE response to NES detectable in the serum of naïve mice while the serum after primary *Nb* infection showed a faint staining for secretions of adult worms (L5) and preparations of whole worm homogenates at ~100 kDa and above 180 kDa (**Figure 1B** and **Supplementary Figure S3**). This may indicate that only some antigen multimers are detected by low-affinity antibodies after primary infection. However, a discrete and overlapping set of NES antigens was recognized by both IgG1 and IgE antibodies from secondary *Nb*-infected mice with a broad signal between 45 and 55 kDa and additional signals at 70 kDa and above 180 kDa (**Figure 1B**). Importantly, this discrete band pattern was only detectable in non-reducing conditions which preserves inter- and intramolecular disulfide bonds of the proteins. We then further separated NES proteins by size and charge using two-dimensional gel electrophoresis (2D SDS-PAGE) followed by Western blotting to reveal the complexity of the detected NES antigens. We identified several spots at about 55 or 70 kDa separated by the pH gradient suggesting that the detected antigens consist of numerous proteins with similar size but different charge (**Figure 1C**). Interestingly, the 70 kDa spots basically mirrored the charge-based distribution of the 55 kDa spots. This may indicate differences in glycosylation although further analysis would be required to confirm this assumption.

### Identification of a new venom/allergen-like protein family in NES of L3 larvae

**Figure 2:**
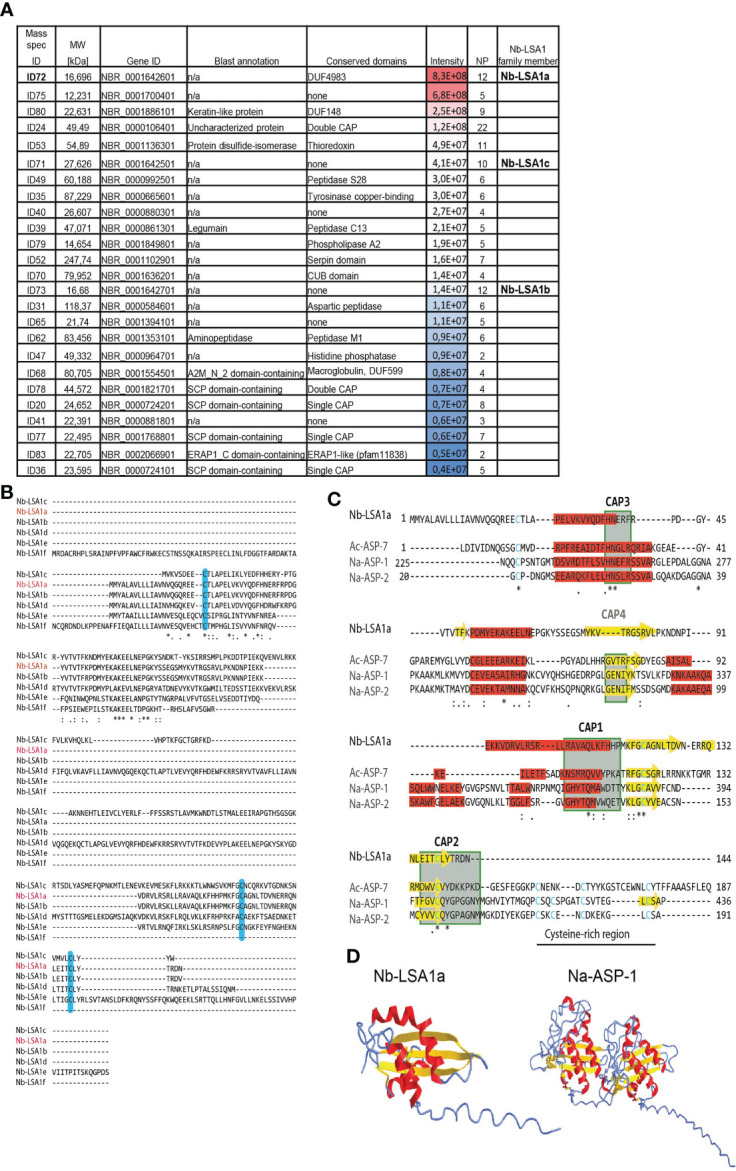
Top 25 most abundant proteins in the secretory proteins of *Nb* L3 stage larvae (NES). **(A)** Table summarizes the top 25 most abundant proteins in NES, representative for 2 separate LC-MS/MS runs. Mass spec identification numbers (ID) were assigned to distinguish between different proteins. Gene ID is taken from WormBase ParaSite database after Blast search for *Nb* genome annotations (Taxonomy ID 27835). Conserved domains were identified using PfamScan (EMBL-EBI) and CD-Search (from NCBI). Peptide intensity score is visualized by color code. MW, Molecular Weight; NP, Number of unique peptides; n/a, not available. **(B)** Multiple sequence alignment of five Nb-LSA1a paralogues. The deduced amino acid sequence of the NBR_0001642601 gene, assigned Nb-LSA1a (red letters), was aligned to its five paralogues, using Clustal Omega. The following consensus symbols are used for amino acid alignment: `*´ indicates identical alignment, `:´ indicates that substitutions are conserved, `.´ means weak similarity of substitutions; Cysteine residues are shaded in light blue. **(C)** Comparison of Nb-LSA1a sequence with selected CAP-domain proteins of known structure. Alignment of Nb-LSA1a with Ac-ASP-7 (PDB entry 3s6s), Na-ASP-1 (PDB entry 3nt8) and Na-ASP-2 (PDB entry 1u53). CAP domains 1-4 are shown in green boxes, Cysteine residues are shown in blue. The Cysteine-rich region, not present in Nb-LSA1a, is indicated by a black line. The same consensus symbols for amino acid alignment as in B were used. Sequence alignments were generated manually based on initial Clustal Omega prediction, secondary structures are shaded in red (α-helix) and yellow (β-strand) according to AlphaFold Protein Structure Prediction. **(D)** AlphaFold prediction and experimental structure for Nb-LSA1a and Na-ASP-1 with the same color-coding as in C. * indicates identitical alignment and ** or *** therefore simply means that two or three identical alignments are next to each other.

To further analyze NES components and identify individual antigens, we performed Liquid Chromatography Mass Spectrometry (LC-MS/MS) of eluted gel slices in the area of interest based on the Western blot analysis. We identified a total of 76 proteins, of which the top 25 most abundant proteins are listed in **Figure 2A**. Only three proteins showed a match with already described proteins exhibiting peptidase activity (legumain, aminopeptidase), as well as a protein disulfide-isomerase, while all others were uncharacterized proteins. Some of the uncharacterized proteins contained domains found in serine proteases (PF05577), histidine phosphatases (PF00328), copper-binding tyrosinase (PF01549), or macroglobulin (PF01835). ID68, a macroglobulin-related protein, may confer endopeptidase inhibitor activity. ID52 contains a Serpin domain (PF00079), characteristic for serine protease inhibitors whose role in nematodes is still poorly defined. ID70 contains a CUB domain (PF00431) often found in peptidases. The most frequently represented group of proteins in the NES products belonged to the SCP/TAPS superfamily (19). This superfamily also contains members of the Venom Allergen-Like (VAL) or Ancylostoma Secreted Protein (ASP) families, which are very abundant in helminth secretions (20, 21). However, molecular targets and functions remain largely elusive. The core of helminth VALs consists of CAP domains with characteristic Cysteine-rich regions (PF00188). Blast annotation and domain analysis showed that at least five proteins contained single or double CAP-domains. Interestingly, one of the proteins (ID78) with a single CAP domain is closely related to *C. elegans* Venom-Allergen-like protein 1 (vap-1).

ID72 was the most abundant protein in all NES preparations. BLAST search against the *Nb* genome on the WormBase ParaSite database revealed that this 16,696 kDa protein with 144 amino acids (aa) is encoded by the gene NBR_0001642601 with seven exons. Additionally, this gene has 5 uncharacterized paralogues in the *Nb* genome, two of which were also detected in our LC-MS/MS analysis (**Figure 2A**). We termed this protein family Nb-LSA1 for “*Nippostrongylus brasiliensis* larval secreted protein 1”, and assigned Nb-LSA1a to ID72. Then, Clustal Omega (22) was used to align the Nb-LSA1a protein sequence with the other 5 family members (**Figure 2B**). Nb-LSA1a is most closely related to Nb-LSA1b (97% protein sequence identity and same size, encoded by the gene NBR_0001642701). The protein sequence identity of Nb-LSA1a to the other family members is only 19-29%. The sizes of these proteins are: 27,6 kDa (Nb-LSA1c, encoded by NBR_0001642501), 35,8 kDa (Nb-LSA1d, encoded by NBR_0001642801), 23,1 kDa (Nb-LSA1e, encoded by NBR_0002055701) and 17,2 kDa (Nb-LSA1f, encoded by NBR_0000291501). A signal peptide motif (first 17-aa) is only present in four family members and missing in Nb-LSA1c and Nb-LSA1f.

The sequence of the initially identified protein Nb-LSA1a was then used to search for homologues in other nematode species using the SWISS-MODEL database (23). A sequence similarity of 21,15% was found for the dog hookworm protein Ac-ASP-7, and 14,29% similarity for the human hookworm protein Na-ASP-1. Although the algorithm used for conserved domain search in **Figure 2A** did not identify a CAP motif for Nb-LSA1a, the result of the homology analysis and the known sequence diversity of the CAP domains supported the idea that Nb-LSA1a might indeed contain a CAP domain. To investigate this more closely, Nb-LSA1a was subjected to comparative analysis with ASPs of known structure, namely Ac-ASP-7 (PDB entry 3s6s), Na-ASP-1 (PDB entry 3nt8) and Na-ASP-2 (PDB entry 1u53). By comparing the sequence and structural features, conserved CAP sequence motifs could be identified in Nb-LSA1a (**Figure 2C**).

The CAP motifs CAP1, CAP2 and CAP3, which are relatively well conserved between the so far known CAP domain-containing proteins, are also present in Nb-LSA1a. Sequence alignment furthermore showed that Nb-LSA1a does not contain a CAP4 motif and is also missing the cysteine-rich region. This region is only weakly conserved and is not a central component for the 3D-structure of the CAP domain. Furthermore, the most likely structure for Nb-LSA1a was generated using AlphaFold prediction algorithm (24) and compared to the known crystal structure of Na-ASP-1 which is composed of two CAP domains (**Figure 2D**). The accuracy of our AlphaFold model was scored as highly accurate with a predicted local distance difference test (pLDDT) >90% (**Supplementary Figure S4**). The arrangement of α-helices and β-strands of Nb-LSA1a clearly resembles one CAP domain of Na-ASP-1. Therefore, it appears that Nb-LSA1a is a CAP domain protein.

### Recombinantly expressed Nb-LSA1a is recognized by immune serum of Nb-infected mice

**Figure 3:**
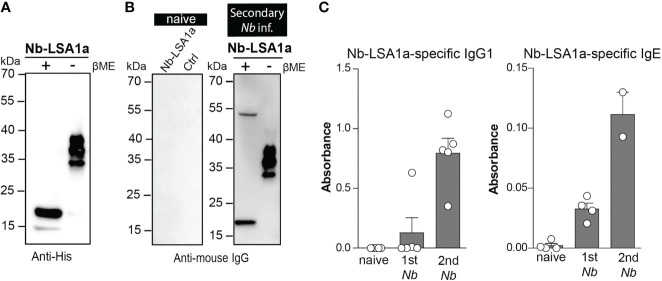
Native Nb-LSA1a forms oligomers and is detectable by serum IgG1 and IgE from secondary *Nb*-infected mice. **(A)** Detection of purified His-tagged Nb-LSA1a from supernatant of transfected HEK293T cells after SDS-PAGE under reducing (+) and non-reducing **(-)** conditions and Western blot using an anti-His-tag antibody. **(B)** Supernatant of HEK293T cells expressing Nb-LSA1-His or empty His vector (Ctrl) was analyzed by standard SDS-PAGE under reducing (+) and non-reducing **(-)** conditions and Western blotting with serum from naïve (left) or secondary *Nb-*infected mice (right), following by detection with an anti-mouse IgG antibody. **(C)** Detection of Nb-LSA1a-specific IgG1 (left) and IgE (right) in serum from naïve, primary (1st) or secondary (2nd) *Nb-*infected mice. Bars show the mean+SEM with 4-5 mice per group.

To further characterize the immunogenicity of Nb-LSA1a, we expressed a C-terminally His-tagged version in HEK293T cells and first performed Western blot analysis of supernatants with anti-His antibodies. Under reducing conditions (+β-ME) Nb-LSA1a appeared as a dominant band of approximately 17 kDa (**Figure 3A**). However, under non-reducing conditions (-β-ME), the 17 kDa band was almost gone and three other bands at around 30-40 kDa appeared (**Figure 3A**). This suggests that Nb-LSA1a is actually expressed as dimer/trimer.

Next, we addressed the question whether Nb-LSA1a is indeed recognized by immune serum from *Nb*-infected mice. As expected, no bands appeared when blots were hybridized with serum from naïve mice. In contrast, serum isolated from mice after secondary *Nb* infection showed basically the same staining pattern as the anti-His antibodies (**Figure 3B**). ELISA analysis further revealed that Nb-LSA1a-specific IgG1 and IgE is generated in *Nb*-infected mice and both antibody levels increased after secondary as compared to primary infection (**Figure 3C**). Based on the strong humoral immune response against Nb-LSA1a we further investigated whether immunization of mice with Nb-LSA1a could protect against *Nb* infection.

### Immunization with Nb-LSA1a elicits basophil-dependent protective immunity

**Figure 4:**
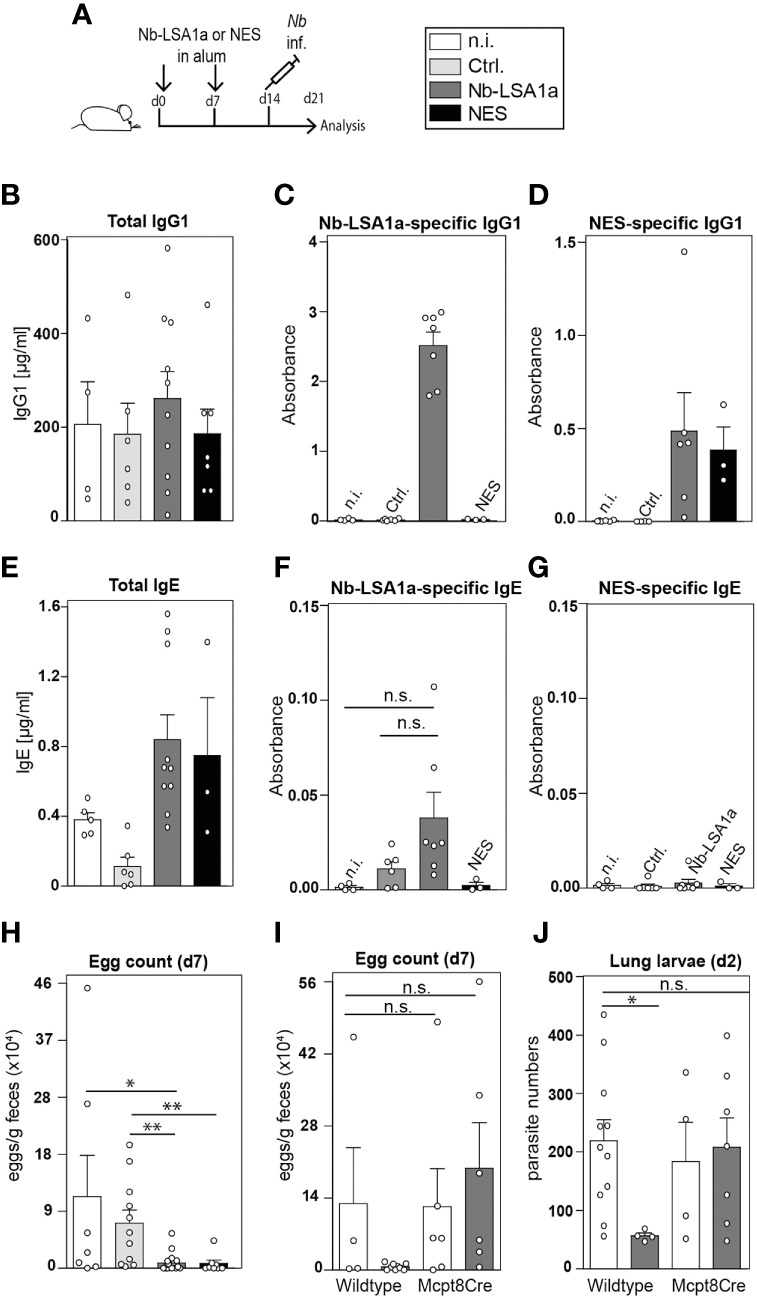
Basophil-mediated protection in Nb-LSA1a immunized mice. **(A)** Experimental setup. Mice were immunized with 1-10 µg protein (NES, Nb-LSA1a or Ctrl) in alum i.p. on day 0 and 7, infected with *Nb* on day 14 and analyzed on day 21. **(B–D)** Total **(B)**, Nb-LSA1a-specific **(C)**, and NES-specific **(D)** serum IgG1 levels of wild-type mice which had not been immunized (n.i., white bar), or immunized with supernatant of HEK293T cells transfected either with an empty vector (Ctrl., light gray bar) or Nb-LSA1a vector (dark gray bar), or immunized with NES (black bar). **(E–G)** Total **(E)**, Nb-LSA1a-specific **(F)**, and NES-specific **(G)** serum IgE levels of wild-type mice, treated as described in B-D. Calculation of mouse serum concentrations for 4 to 10 samples and absorbance calculated for 4 to 7 samples per group. **(H, I)** Fecal egg counts on day 7 post *Nb* infection. **(I)** Immunization was carried out with purification buffer (white bars) or Nb-LSA1a purified protein (dark grey bar). **(J)** Number of larvae in the lung on day 2 post *Nb* infection of wild-type or basophil-deficient Mcpt8Cre mice, immunized with purification buffer (white bars) or Nb-LSA1a purified protein (dark gray bar). Data shown are combined from four experiments with each of 3-6 individual mice per group **(H)** and two experiments with 3 **(I)** or 3-5 **(J)** mice per group. Statistical analysis was performed with Mann-Whitney U test (*P<0.05, **P<0.01). n.s. = not significant.

To determine whether immunization of mice with Nb-LSA1a is sufficient to protect against *Nb* infection we performed experiments using a standard intraperitoneal immunization protocol with alum adjuvant (**Figure 4A**). In brief, mice were immunized with Nb-LSA1a or NES in alum on day 0 and 7, infected with *Nb* on day 14 and analyzed 7 days after infection. While *Nb* infection elicited similar levels of total IgG1 serum antibodies in all groups of mice, Nb-LSA1a-specific IgG1 was only present in the serum of Nb-LSA1a-immunized mice (**Figure 4B, C**). IgG1 in the serum of Nb-LSA1a-immunized mice also bound to NES-coated plates, which confirms that Nb-LSA1a is a prominent antigen in the whole secreted protein mixture (**Figure 4D**). Unexpectedly, we did not detect a significant increase of anti-Nb-LSA1a IgE (**Figures 4E–G**).

Importantly, Nb-LSA1a-immunized mice showed strongly reduced egg burden in fecal pellets, similar to NES-immunized mice (**Figure 4H**). Previous studies have shown that basophils contribute to protection against secondary *Nb* infection. Hence, we decided to compare the protective effect of Nb-LSA1a immunization in wild-type and basophil-deficient Mcpt8Cre mice (15). Egg counts in fecal pellets of Nb-LSA1a-immunized Mcpt8Cre mice were similar to egg counts from non-immunized wild-type or Mcpt8Cre mice (**Figure 4I**). This was not due to an impaired anti-Nb-LSA1a IgG1 response in Mcpt8Cre mice (data not shown). To further analyze whether this protective basophil-mediated effect occurs already in the skin as the first anatomical site of infection, we determined the number of larvae that reached the lung on day 2 after infection. While non-immunized wild-type or Mcpt8Cre mice contained about 200 larvae, this number was reduced to about 50 larvae only in Nb-LSA1a-immunized wild-type mice (**Figure 4J**).

Overall, these data indicate that immunization with Nb-LSA1a, a newly identified secreted protein of *Nb* L3 larvae, elicits a strong IgG1 response and provides basophil-mediated protective immunity against *Nb* infection mainly in the skin or before they reach the lung. This finding illustrates that secreted proteins of the free-living larval stage can have important and yet to be determined functions for migration and survival within the infected host.

## Discussion

Development of efficient and safe vaccines against hookworm infections remains a major challenge. Such vaccines would reduce disease burden and ameliorate clinical conditions even if achievement of sterile immunity is probably not realistic (6, 25). Detailed understanding of the mechanisms how hookworms establish their parasitic niches and how the immune system responds to infection is detrimental to develop new vaccination strategies. Basic research using mouse models of hookworm infections such as infections of mice with *Nb* or *Heligmosomoides polygyrus (Hp)* can be helpful in this regard (26). For example, the mechanisms of worm expulsion from the intestine by IL-13-elicited and STAT6-dependent activation of goblet cells and smooth muscle cells are quite well understood (27, 28). However, details such as the role of tuft cells, ILC2s and alternatively activated macrophages are constantly emerging (12, 29, 30). In the present study, we sought to identify and characterize new *Nb*-derived antigens that elicit a humoral immune response and provide protection against *Nb* infection.

It is well established that *Nb* or *Hp* infections of mice elicit a strong germinal center response and elevations of serum IgG1 and IgE levels. However, primary infections induce an antibody response with very few somatic mutations which might explain the lack of detectable NES-specific antibodies by ELISA or Western blot (31–34). Here, we also report high levels of IgE and IgG1 in the serum of *Nb*-infected C57BL/6 mice after primary infection and a further increase after secondary infection.

When analyzing the reactivity of induced antibodies towards parasite antigens (secreted as well as whole worm extracts), we only detected NES-reactive antibodies after secondary infection by ELISA.

These results indicate that *Nb*-specific IgE and IgG1 antibodies with germinal center-dependent affinity maturation are only induced after repeated infections. This assumption is also supported by a previous study which reported the identification of a *Nb*-derived antigen recognized by a monoclonal IgE antibody without somatic mutations (32). McCoy et al. further demonstrated that primary *Hp* infection is accompanied by production of antibodies with irrelevant specificities while parasite-specific antibodies only arise after multiple infections (34). One might assume that complex pathogens such as helminths express a large variety of antigens. However, we repeatedly detected a very restricted set of antigens in NES of L3 larvae that was recognized by antibodies from secondary *Nb*-infected mice at a size around 55 or 70 kDa.

When analyzing the antigen specificity of polyclonal antibody response for *Hp* infections it has been shown before that HES elicits an antibody response directed against restricted glycan and peptide epitopes (35). Interestingly, in line with our findings they also observed that this response is directed at secreted, rather than whole worm products. Immunization with three secreted SCP/TAPS proteins of adult *Hp* worms elicited protective IgG1-dependent but basophil-independent immunity by more efficient larval trapping in the submucosa of the small intestine (36). We used NES from L3 stage larvae, the infective larval stage, to screen for serum reactivity because we reasoned that humoral immunity against the first encountered antigens secreted by L3 larvae during skin invasion could lead us to identification of critical proteins required for successful parasitism.

Using LC-MS/MS analysis, we identified a new subfamily of SCP/TAPS proteins with 6 members in the *Nb* genome (Nb-LSA1a-f). Interestingly, the SCP/TAPS superfamily, members of which are also named VAL and ASP proteins, is very abundant in the human hookworm *N. americanus* and other parasitic nematodes but not in free-living nematodes (5). Previous proteomic analysis that compared the secretome of L3 larvae and adult worms from *Nb* already noticed the abundance of SCP/TAPS proteins in the secretomes (37).This suggests that SCP/TAPS proteins play a role in host infection and/or evasion from rapid elimination by the immune system. However, the biological functions and properties of these proteins remain elusive (4).

Nb-LSA1a was the most abundant protein with the highest signal intensity in all LC-MS/MS runs. Nb-LSA1a shares 21% sequence identity with *A. caninum* Ancylostoma-secreted protein (Ac-ASP-7) and 14% sequence identity with *N. americanus* Ancylostoma-secreted protein 1 (Na-ASP-1). The basis for development of vaccines was set in the field of canine hookworm infections. Here, the discovery that radiation-attenuated *A*. *caninum* L3 larval vaccine protected against challenge infection led to identification of the *Ancylostoma*-secreted proteins (ASPs) which belong to the SCP/TAPS superfamily (38). Such ASP proteins from *N. americanus* turned out to be a promising class of antigens from infective L3 larvae and were tested as potential human anti-hookworm vaccines (39). One potential vaccine candidate was indeed Na-ASP-2 that provided significant protection against challenge infections but at the same time data from a clinical trial in a hookworm-endemic area showed that it resulted in generalized IgE-elicited urticarial reactions (40). In fact, Na-ASP-2-specific IgE is readily detectable in serum of people living in endemic areas. Therefore, Na-ASP-2 was not further considered and other vaccine candidates are currently under investigation, especially a combination vaccine with Na-GST-1, a glutathione-S-transferase, and Na-APR-1, a aspartic protease modified to lack protease activity (6). More recently, a phase I trial with ultraviolet C (UVC)-attenuated *N. americanus* L3 larvae was successfully completed (41). However, vaccination with defined recombinantly expressed proteins has obvious advantages with regard to vaccine production at large scales.

Our study shows that immunization of mice with Nb-LSA1a elicits a strong antigen-specific IgG1 response but no detectable antigen-specific IgE. This was surprising because anti-Nb-LSA1a IgE is clearly detectable in serum of *Nb* infected mice. One explanation would be that the quality of the humoral immune response elicited by immunization versus infection is different. Alternatively, the larger amounts of IgG1 antibodies in the serum of immunized mice may cover all epitopes on Nb-LSA1a and thereby prevent binding of IgE antibodies in ELISA and Western blot analysis. In any case, the *Nb* infection of immunized mice did not result in severe local or systemic allergic reactions. It has been shown before, that IgG1 antibodies activate macrophages during vaccination or infection of mice with the helminth *Heligmosomoides polygyrus bakeri* and these macrophages probably contribute to protection (36, 42). Therefore, we will further investigate whether Nb_LSA1a-specific IgG1 activates macrophages in the skin which could be one component of protective immunity. Importantly, the transition of L3 larvae from skin to lung was severely impaired in immunized mice and this protective effect was lost in basophil-deficient mice. Basophils have been recognized before to confer protection in the skin against secondary *Nb* infection (9). However, the critical antigens that elicit basophil-mediated protection in the skin remained unclear. We fill this gap of knowledge by showing that immunization with a single secreted protein, Nb-LSA1a, is sufficient to strongly reduce larval migration to the lung in a basophil-dependent manner. As a consequence this effect resulted in severely reduced fecal egg counts. Basophils are a major source of vasoactive substances, proteases, lipid mediators, chemokines, and Th2-associated cytokines such as IL-4 and IL-5 that promote accumulation of alternatively activated macrophages (AAM) and eosinophils in the skin (9, 43). Further studies are needed to characterize the function of basophils in human skin and to identify new secreted antigens from L3 stage larvae of human hookworms that elicit a strong IgG1 and a weak IgE response. Development of efficient hookworm vaccines that prevent larval migration from skin to lung seems possible and would provide a great benefit for millions of people living in hookworm-endemic regions.

## Data availability statement

The data presented in the study have been deposited to the ProteomeXchange Consortium via the PRIDE partner repository with the identifier PXD035568. Further inquiries can be directed to the corresponding authors.

## Ethics statement

The animal study was reviewed and approved by Government of Lower Franconia.

## Author contributions

NT and DV designed experiments. NT, DD, LR, DM and DR performed experiments. NT, DR, LR, BW and HS analysed data. DV and BW acquired funding. All authors contributed to the article and approved the submitted version.

## Funding

This work was supported by the Deutsche Forschungsgemeinschaft (DFG) grants TRR130_TP20 and RTG1660_B3 to DV, and TRR130_C02 to BW.

## Acknowledgments

We thank Kirstin Castiglione for technical support and members of the Voehringer group for helpful discussions.

## Conflict of interest

The authors declare that the research was conducted in the absence of any commercial or financial relationships that could be construed as a potential conflict of interest.

## Publisher’s note

All claims expressed in this article are solely those of the authors and do not necessarily represent those of their affiliated organizations, or those of the publisher, the editors and the reviewers. Any product that may be evaluated in this article, or claim that may be made by its manufacturer, is not guaranteed or endorsed by the publisher.

## References

[1] Loukas A Hotez PJ Diemert D Yazdanbakhsh M McCarthy JS Correa-Oliveira R. Hookworm infection. Nat Rev Dis Primers (2016) 2:16088. doi: 10.1038/nrdp.2016.88 27929101

[2] Doyle SR Cotton JA. Genome-wide approaches to investigate anthelmintic resistance. Trends Parasitol (2019) 35(4):289–301. doi: 10.1016/j.pt.2019.01.004 30733094

[3] Schneider B Jariwala AR Periago MV Gazzinelli MF Bose SN Hotez PJ. A history of hookworm vaccine development. Hum Vaccin (2011) 7(11):1234–44. doi: 10.4161/hv.7.11.18443 PMC3323499 22064562

[4] Logan J Pearson MS Manda SS Choi YJ Field M Eichenberger RM. Comprehensive analysis of the secreted proteome of adult necator americanus hookworms. PloS Negl Trop Dis (2020) 14(5):e0008237. doi: 10.1371/journal.pntd.0008237 32453752 PMC7274458

[5] Tang YT Gao X Rosa BA Abubucker S Hallsworth-Pepin K Martin J. Genome of the human hookworm necator americanus. Nat Genet (2014) 46(3):261–9. doi: 10.1038/ng.2875 PMC3978129 24441737

[6] Hotez PJ Diemert D Bacon KM Beaumier C Bethony JM Bottazzi ME. The human hookworm vaccine. Vaccine (2013) 31 Suppl 2:B227–32. doi: 10.1016/j.vaccine.2012.11.034 PMC3988917 23598487

[7] Kim DG Park JH Kim JL Jung BK Jeon SJ Lim H. Intestinal nematodes from small mammals captured near the demilitarized zone, gyeonggi province, republic of Korea. Korean J Parasitol (2015) 53(1):135–9. doi: 10.3347/kjp.2015.53.1.135 PMC4384801 25748722

[8] Harvie M Camberis M Tang SC Delahunt B Paul W Le Gros G. The lung is an important site for priming CD4 T-cell-mediated protective immunity against gastrointestinal helminth parasites. Infect Immun (2010) 78(9):3753–62. doi: 10.1128/IAI.00502-09 PMC2937440 20605978

[9] Obata-Ninomiya K Ishiwata K Tsutsui H Nei Y Yoshikawa S Kawano Y. The skin is an important bulwark of acquired immunity against intestinal helminths. J Exp Med (2013) 210(12):2583–95. doi: 10.1084/jem.20130761 PMC3832932 24166714

[10] Chen F El-Naccache DW Ponessa JJ Lemenze A Espinosa V Wu W. Helminth resistance is mediated by differential activation of recruited monocyte-derived alveolar macrophages and arginine depletion. Cell Rep (2022) 38(2):110215. doi: 10.1016/j.celrep.2021.110215 35021079 PMC9403845

[11] Schwartz C Turqueti-Neves A Hartmann S Yu P Nimmerjahn F Voehringer D. Basophil-mediated protection against gastrointestinal helminths requires IgE-induced cytokine secretion. Proc Natl Acad Sci USA (2014) 111(48):E5169–77. doi: 10.1073/pnas.1412663111 PMC4260590 25404305

[12] Krljanac B Schubart C Naumann R Wirtz S Culemann S Kronke G. RELMalpha-expressing macrophages protect against fatal lung damage and reduce parasite burden during helminth infection. Sci Immunol (2019) 4(35). doi: 10.1126/sciimmunol.aau3814 31126996

[13] Chen F Wu W Millman A Craft JF Chen E Patel N. Neutrophils prime a long-lived effector macrophage phenotype that mediates accelerated helminth expulsion. Nat Immunol (2014) 15(10):938–46. doi: 10.1038/ni.2984 PMC4479254 25173346

[14] Brindley PJ Dobson C. Specificity of passive serum protection against nippostrongylus brasiliensis and nematospiroides dubius in mice. Aust J Exp Biol Med Sci (1983) 61(Pt 1):37–45. doi: 10.1038/icb.1983.4 6870676

[15] Ohnmacht C Schwartz C Panzer M Schiedewitz I Naumann R Voehringer D. Basophils orchestrate chronic allergic dermatitis and protective immunity against helminths. Immunity (2010) 33(3):364–74. doi: 10.1016/j.immuni.2010.08.011 20817571

[16] Perez-Riverol Y Bai J Bandla C Garcia-Seisdedos D Hewapathirana S Kamatchinathan S. The PRIDE database resources in 2022: a hub for mass spectrometry-based proteomics evidences. Nucleic Acids Res (2022) 50(D1):D543–52. doi: 10.1093/nar/gkab1038 PMC8728295 34723319

[17] Jumper J Evans R Pritzel A Green T Figurnov M Ronneberger O. Highly accurate protein structure prediction with AlphaFold. Nature (2021) 596(7873):583–9. doi: 10.1038/s41586-021-03819-2 PMC8371605 34265844

[18] Varadi M Anyango S Deshpande M Nair S Natassia C Yordanova G. AlphaFold protein structure database: massively expanding the structural coverage of protein-sequence space with high-accuracy models. Nucleic Acids Res (2022) 50(D1):D439–d444. doi: 10.1093/nar/gkab1061 34791371 PMC8728224

[19] Cantacessi C Campbell BE Visser A Geldhof P Nolan MJ Nisbet AJ. A portrait of the "SCP/TAPS" proteins of eukaryotes–developing a framework for fundamental research and biotechnological outcomes. Biotechnol Adv (2009) 27(4):376–88. doi: 10.1016/j.biotechadv.2009.02.005 19239923

[20] Wilbers RHP Schneiter R Holterman MHM Drurey C Smant G Asojo OA. Secreted venom allergen-like proteins of helminths: Conserved modulators of host responses in animals and plants. PloS Pathog (2018) 14(10):e1007300. doi: 10.1371/journal.ppat.1007300 30335852 PMC6193718

[21] Viney M. The genomic basis of nematode parasitism. Brief Funct Genomics (2018) 17(1):8–14. doi: 10.1093/bfgp/elx010 28472353 PMC5886223

[22] Sievers F Wilm A Dineen D Gibson TJ Karplus K Li W. Fast, scalable generation of high-quality protein multiple sequence alignments using clustal omega. Mol Syst Biol (2011) 7:539. doi: 10.1038/msb.2011.75 21988835 PMC3261699

[23] Waterhouse A Bertoni M Bienert S Studer G Tauriello G Gumienny R. SWISS-MODEL: homology modelling of protein structures and complexes. Nucleic Acids Res (2018) 46(W1):W296–303. doi: 10.1093/nar/gky427 PMC6030848 29788355

[24] Senior AW Evans R Jumper J Kirkpatrick J Sifre L Green T. Improved protein structure prediction using potentials from deep learning. Nature (2020) 577(7792):706–10. doi: 10.1038/s41586-019-1923-7 31942072

[25] Miller TA. Industrial development and field use of the canine hookworm vaccine. Adv Parasitol (1978) 16:333–42. doi: 10.1016/s0065-308x(08)60577-1 364958

[26] Gause W. The immune response to parasitic helminths: insights from murine models. Trends Immunol (2003) 24(5):269–77. doi: 10.1016/s1471-4906(03)00101-7 12738422

[27] Katona IM Urban JF Jr. Finkelman FD. The role of L3T4+ and lyt-2+ T cells in the IgE response and immunity to nippostrongylus brasiliensis. J Immunol (1988) 140(9):3206–11. 2966208

[28] Urban JF Jr. Noben-Trauth N Donaldson DD Madden KB Morris SC Collins M. IL-13, IL-4Ralpha, and Stat6 are required for the expulsion of the gastrointestinal nematode parasite nippostrongylus brasiliensis. Immunity (1998) 8(2):255–64. doi: 10.1016/s1074-7613(00)80477-x 9492006

[29] Neill DR Wong SH Bellosi A Flynn RJ Daly M Langford TK. Nuocytes represent a new innate effector leukocyte that mediates type-2 immunity. Nature (2010) 464(7293):1367–70. doi: 10.1038/nature08900 PMC2862165 20200518

[30] von Moltke J Ji M Liang HE Locksley RM. Tuft-cell-derived IL-25 regulates an intestinal ILC2-epithelial response circuit. Nature (2016) 529(7585):221–5. doi: 10.1038/nature16161 PMC4830391 26675736

[31] Turqueti-Neves A Otte M Schwartz C Schmitt ME Lindner C Pabst O. The extracellular domains of IgG1 and T cell-derived IL-4/IL-13 are critical for the polyclonal memory IgE response *in vivo* . PloS Biol (2015) 13(11):e1002290. doi: 10.1371/journal.pbio.1002290 26523376 PMC4629909

[32] Pochanke V Koller S Dayer R Hatak S Ludewig B Zinkernagel RM. Identification and characterization of a novel antigen from the nematode nippostrongylus brasiliensis recognized by specific IgE. Eur J Immunol (2007) 37(5):1275–84. doi: 10.1002/eji.200737135 17429848

[33] Kanayama N HC Magari M Ohtani K Hikida M Yamada M Matsuda S. Use of secondarily revised VH genes in IgE antibodies produced in mice infected with the nematode nippostrongylus brasiliensis. Immunol Lett (2001) 77(3):181–6. doi: 10.1016/s0165-2478(01)00216-4 11410252

[34] McCoy KD Stoel M Stettler R Merky P Fink K Senn BM. Polyclonal and specific antibodies mediate protective immunity against enteric helminth infection. Cell Host Microbe (2008) 4(4):362–73. doi: 10.1016/j.chom.2008.08.014 18854240

[35] Hewitson JP Filbey KJ Grainger JR Dowle AA Pearson M Murray J. Heligmosomoides polygyrus elicits a dominant nonprotective antibody response directed against restricted glycan and peptide epitopes. J Immunol (2011) 187(9):4764–77. doi: 10.4049/jimmunol.1004140 PMC4306209 21964031

[36] Hewitson JP Filbey KJ Esser-von Bieren J Camberis M Schwartz C Murray J. Concerted activity of IgG1 antibodies and IL-4/IL-25-dependent effector cells trap helminth larvae in the tissues following vaccination with defined secreted antigens, providing sterile immunity to challenge infection. PloS Pathog (2015) 11(3):e1004676. doi: 10.1371/journal.ppat.1004676 25816012 PMC4376884

[37] Sotillo J Sanchez-Flores A Cantacessi C Harcus Y Pickering D Bouchery T. Secreted proteomes of different developmental stages of the gastrointestinal nematode nippostrongylus brasiliensis. Mol Cell Proteomics (2014) 13(10):2736–51. doi: 10.1074/mcp.M114.038950 PMC4188999 24994561

[38] Mendez S Zhan B Goud G Ghosh K Dobardzic A Wu W. Effect of combining the larval antigens ancylostoma secreted protein 2 (ASP-2) and metalloprotease 1 (MTP-1) in protecting hamsters against hookworm infection and disease caused by ancylostoma ceylanicum. Vaccine (2005) 23(24):3123–30. doi: 10.1016/j.vaccine.2004.12.022 15837211

[39] Bethony J Loukas A Smout M Brooker S Mendez S Plieskatt J. Antibodies against a secreted protein from hookworm larvae reduce the intensity of hookworm infection in humans and vaccinated laboratory animals. FASEB J (2005) 19(12):1743–5. doi: 10.1096/fj.05-3936fje 16037096

[40] Diemert DJ Pinto AG Freire J Jariwala A Santiago H Hamilton RG. Generalized urticaria induced by the Na-ASP-2 hookworm vaccine: implications for the development of vaccines against helminths. J Allergy Clin Immunol (2012) 130(1):169–76.e6. doi: 10.1016/j.jaci.2012.04.027 22633322

[41] Chapman PR Webster R Giacomin P Llewellyn S Becker L Pearson MS. Vaccination of human participants with attenuated necator americanus hookworm larvae and human challenge in Australia: a dose-finding study and randomised, placebo-controlled, phase 1 trial. Lancet Infect Dis (2021) 21(12):1725–36. doi: 10.1016/S1473-3099(21)00153-5 PMC9760122 34419209

[42] Esser-von Bieren J Volpe B Kulagin M Sutherland DB Guiet R Seitz A. Antibody-mediated trapping of helminth larvae requires CD11b and fcgamma receptor I. J Immunol (2015) 194(3):1154–63. doi: 10.4049/jimmunol.1401645 PMC4298127 25548226

[43] Eberle JU Radtke D Nimmerjahn F Voehringer D. Eosinophils mediate basophil-dependent allergic skin inflammation in mice. J Invest Dermatol (2019) 139(9):1957–65 e2. doi: 10.1016/j.jid.2019.03.1129 30910757

