# Supplementary material for: A newly identified secreted larval antigen elicits basophil-dependent protective immunity against N. brasiliensis infection
Source: Front Immunol. 2022 Aug 25;13:979491. doi: 10.3389/fimmu.2022.979491 (PMC9453252; doi:10.3389/fimmu.2022.979491)
Supplement: Supplementary file 1 [file DataSheet_1.pdf]

>NBR_0001642601-mRNA-1 cds: **Nb_LSA1a**

(BamHI) GGATCCATGATGTACGCACTGGCGGTTCTTCTCCTGATCGCGGTCAACGTTCAGGGTCAAAGAGAA

GAGTGCACGCTGGCACCGGAGCTAGTAAAGGTTTATCAAGACTTCCACAACGAACGCTTC

AGACCTGATGGTTATGTCACTGTGACTTTCAAACCTGATATGTACGAGAAAGCTAAAGAA

GAGCTCAATGAACCAGGGAAGTATTCATCGGAGGGAAGTATGTATAAAGTCACTAGGGGC

TCGAGGGTTCTGCCAAAGAACGACAATCCGATCGAGAAAAAGGTGGACAGGGTTTTGAGA

AGCAGGCTCCTGAGGGCAGTTGCTCAACTGAAGTTCCACCATCCTATGAAGTTTGGTTGT

GCCGGAAATCTCACAGATGTTAACGAGAGAAGACAAAATCTCGAAATCACGTGTCTCTAC

ACGCGTGACAACGGGCCC (Apa I)

pcDNA 3.1 (+) C-His:

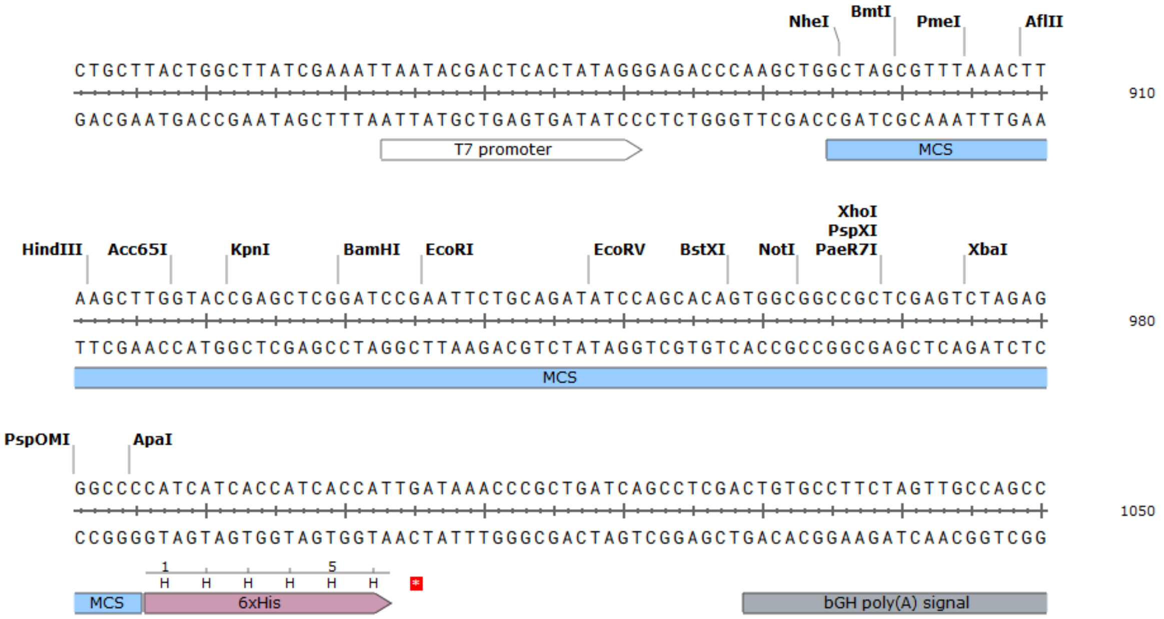

Suppl. Figure 1. Fullenght cDNA of Nb_LSA1a and multiple cloning site of pcDNA3.1 (+) C-His.

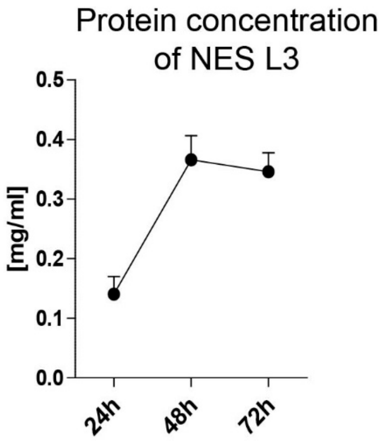

Suppl. Figure 2. Increase of protein concentration in NES of L3 larvae over time. 10,000 L3 stage larvae were cultured in 1 mL 1% glucose in PBS at 37°C and 5% CO_2 and protein concentrations in the supernatant were determined at indicated times by Bradford assay.

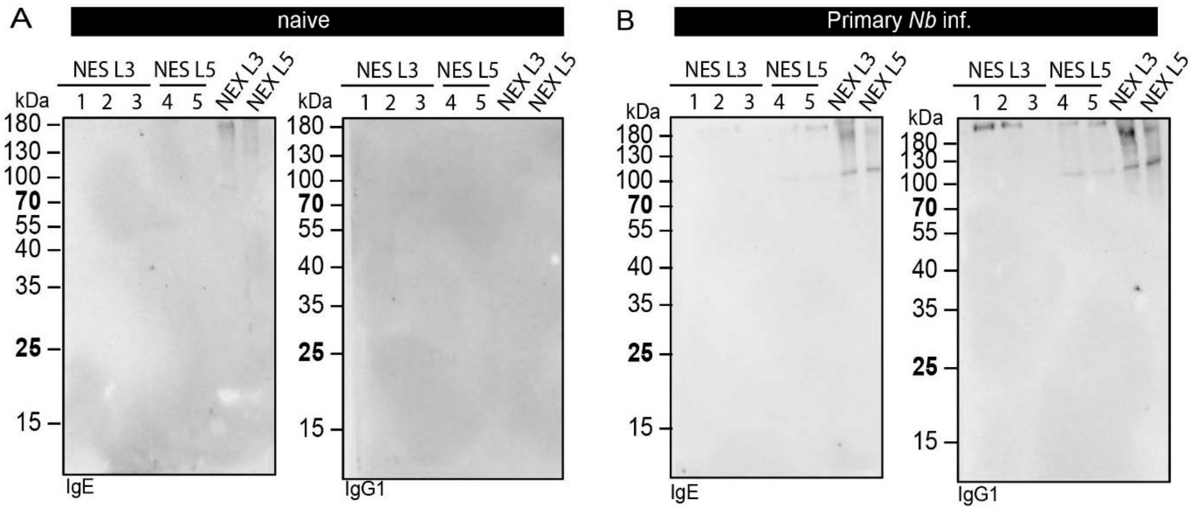

Suppl. Figure 3. Absence of NES-specific antibodies in naïve or primary *Nb*-infected mice. NES and NEX preparations (L3 stage larvae and adults (L5)) were analysed by standard SDS-PAGE under non-reducing conditions and samples were subjected to Western blot (Wb) as indicated. Immunostaining was performed using serum from naïve **(A)** or primary *N. brasiliensis (Nb)* infected mice **(B)**, following detection with either anti-mouse IgE or anti-mouse IgG1. Numbers indicate different batches of prepared *Nb* antigen.

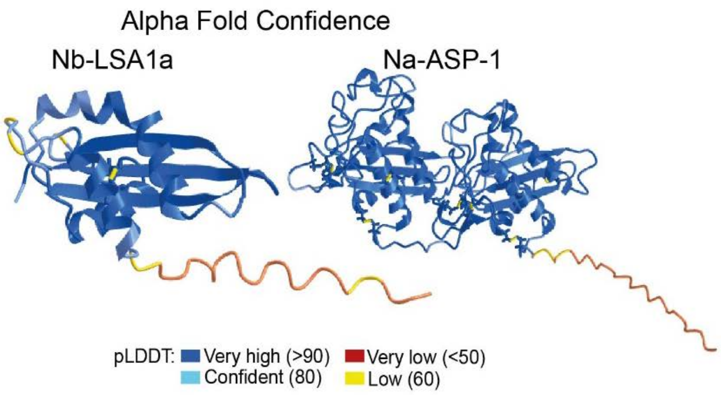

Suppl. Figure 4. AlphaFold prediction and experimental structure for Nb-LSA1a and Na-ASP-1. The prediction is colored by a per-residue confidence metric called predicted local distance difference test (pLDDT) on a scale from 0 to 100.
